# New insights into phenotypic heterogeneity for the distinct lipid accumulation of *Schizochytrium* sp. H016

**DOI:** 10.1186/s13068-022-02126-w

**Published:** 2022-03-25

**Authors:** Zhendong Bao, Yuanmin Zhu, Kai Zhang, Yumei Feng, Meng Zhang, Ruili Li, Longjiang Yu

**Affiliations:** 1 Institute of Resource Biology and Biotechnology, Department of Biotechnology, College of Life Science and Technology, Huazhong University of Science and Technology, No. 1037 Luoyu Road, Wuhan, 430074 China; 2 Key Laboratory of Molecular Biophysics, Ministry of Education, Wuhan, 430074 China; 3 Hubei Engineering Research Center for Both Edible and Medicinal Resources, Wuhan, 430074 China

**Keywords:** *Schizochytrium* sp., Phenotypic heterogeneity, Flow cytometry, Lipid biosynthesis, Transcriptome analysis

## Abstract

**Background:**

*Schizochytrium* sp. is a marine heterotrophic protist and an important sustainable resource for high value-added docosahexaenoic acid in the future. The production of different phenotypes during the continuous subculture of *Schizochytrium* sp. results in a serious reduction in lipid yield and complicates the used of this strain in scientific research and industrial production. Hence, obtaining an improved understanding of the phenotypic differences and molecular mechanisms underlying the cell-to-cell heterogeneity of *Schizochytrium* sp. is necessary.

**Results:**

After continuous culture passage, *Schizochytrium* sp. H016 differentiated into two subpopulations with different morphologies and showed decreased capacity for lipid production. The presence of cell subpopulations with degraded lipid droplets led to a substantial decrease in overall lipid yield. Here, a rapid screening strategy based on fluorescence-activated cell sorting was proposed to classify and isolate subpopulations quickly in accordance with their lipid-producing capability. The final biomass and lipid yield of the subpopulation with high cell lipid content (i.e., H016-H) were 38.83 and 17.22 g/L, respectively, which were 2.07- and 5.38-fold higher than those of the subpopulation with low lipid content (i.e., H016-L), respectively. Subsequently, time‑resolved transcriptome analysis was performed to elucidate the mechanism of phenotypic heterogeneity in different subpopulations. Results showed that the expression of genes related to the cell cycle and lipid degradation was significantly upregulated in H016-L, whereas the metabolic pathways related to fatty acid synthesis and glyceride accumulation were remarkably upregulated in H016-H.

**Conclusion:**

This study innovatively used flow cytometry combined with transcriptome technology to provide new insights into the phenotypic heterogeneity of different cell subpopulations of *Schizochytrium* sp. Furthermore, these results lay a strong foundation for guiding the breeding of oleaginous microorganisms with high lipid contents.

**Supplementary Information:**

The online version contains supplementary material available at 10.1186/s13068-022-02126-w.

## Introduction

Marine microbes have considerable biotechnological potential, and can biosynthesize high value-added single-cell oils to meet the increasing global demand for edible oils and biofuels [1, 2]. Generally, oleaginous microorganisms can synthesize lipids at contents of up to 20%–50% of the cell dry weight and are thus considered as having the greatest potential to be a source of sustainable and low-cost biofuels and chemicals [3, 4]. Microbial oil can be divided into two classes in accordance with fatty acids types, that is, saturated fatty acids (SFAs) and polyunsaturated fatty acids (PUFAs). SFAs such as palmitic acid (C16:0) and stearic acid (C18:0), have 14–20 carbons and are valuable for biodiesel production due to their high oxygen stability and energy density [5, 6]. PUFAs such as eicosapentaenoic acid (EPA, C20:5) and docosahexaenoic acid (DHA, C22:6), have more than 20 carbons and are mainly used as supplements in food and medicine that are considered as essential for human health [7]. In recent years, DHA has attracted extensive attention for its crucial roles in promoting brain development; protecting the retinal; and reducing the risk of some diseases, such as Alzheimer’s disease, schizophrenia, and mood disorders [8].

At present, DHA is commercially produced on a large scale from the marine protist *Schizochytrium* sp. However, in recent years, monocultures under artificial environments, such as specific bioreactors, have been recognized to be capable of exhibiting cell phenotypic heterogeneity. A growing body of evidences shows that microorganisms could evolve adaptively under laboratory preservation conditions [9], or differentiate into subpopulations with different intracellular lipid content [10]. Furthermore, industrial microorganism strains spontaneously degenerate after consecutive subcultivation. This phenomenon would result in changes in morphological and physiological characteristics, thereby greatly reducing the yield of the target product or even resulting in the loss of the capacity for producing desirable chemical compounds [11]. For example, population heterogeneity was observed in the culture of the marine microalga *Nannochloropsis oceanica* IMET1, which had two subpopulations with different lipid synthesis capabilities [12]. The subpopulations with low productivity usually consumed resources during fermentation, thus causing a decline in overall product yield. Therefore, unraveling and reducing phenotypic heterogeneity have become a key aspect of bioprocess optimization.

The phenotypic heterogeneity of industrial microorganisms is inevitable [13]. In the conventional method, excellent strains are commonly screened from single colonies in existing microbial cultures. This method requires a long experimental period and heavy labor while presenting low efficiency. Therefore, it might not obtain strains with high lipid production. The fatty acids that are synthesized by marine oleaginous microorganisms are commonly converted into triacylglycerol (TAG) and stored in lipid droplets [14]. The rapid development of bio-optical sensor technology has enabled the separation of cell subpopulations by using fluorescence-activated cell sorting (FACS) based on signals that are correlated with product abundance [15]. The strategy using Nile red staining to assess the lipid content of single-celled oleaginous microorganisms has become an achievable method, and many studies have demonstrated its capability to reveal cellular heterogeneity [16, 17].

In a previous research, our research group found that the lipid yield of *Schizochytrium* sp. H016 decreased severely during consecutive subculturing and observed cells with two different morphological characteristics. To date, only a few studies have focused on the effects of phenotypic heterogeneity on the fermentation performance of *Schizochytrium* sp. Moreover, no research has been conducted to investigate the rapid sorting of *Schizochytrium* sp. with high lipid content. In this study, a novel efficient procedure was successfully applied for the identification and isolation of heterogeneous *Schizochytrium* sp. subpopulations. The fermentation and physiological characteristics of the two cell subpopulations were further demonstrated to have significant differences in terms of cell division and lipid synthesis capability. Then, sequential transcriptome analysis was performed to provide new insights into cell heterogeneity and physiological behavior.

## Results

### Phenotypic heterogeneity of cell subpopulations in *Schizochytrium* sp. H016

**Fig. 1:**
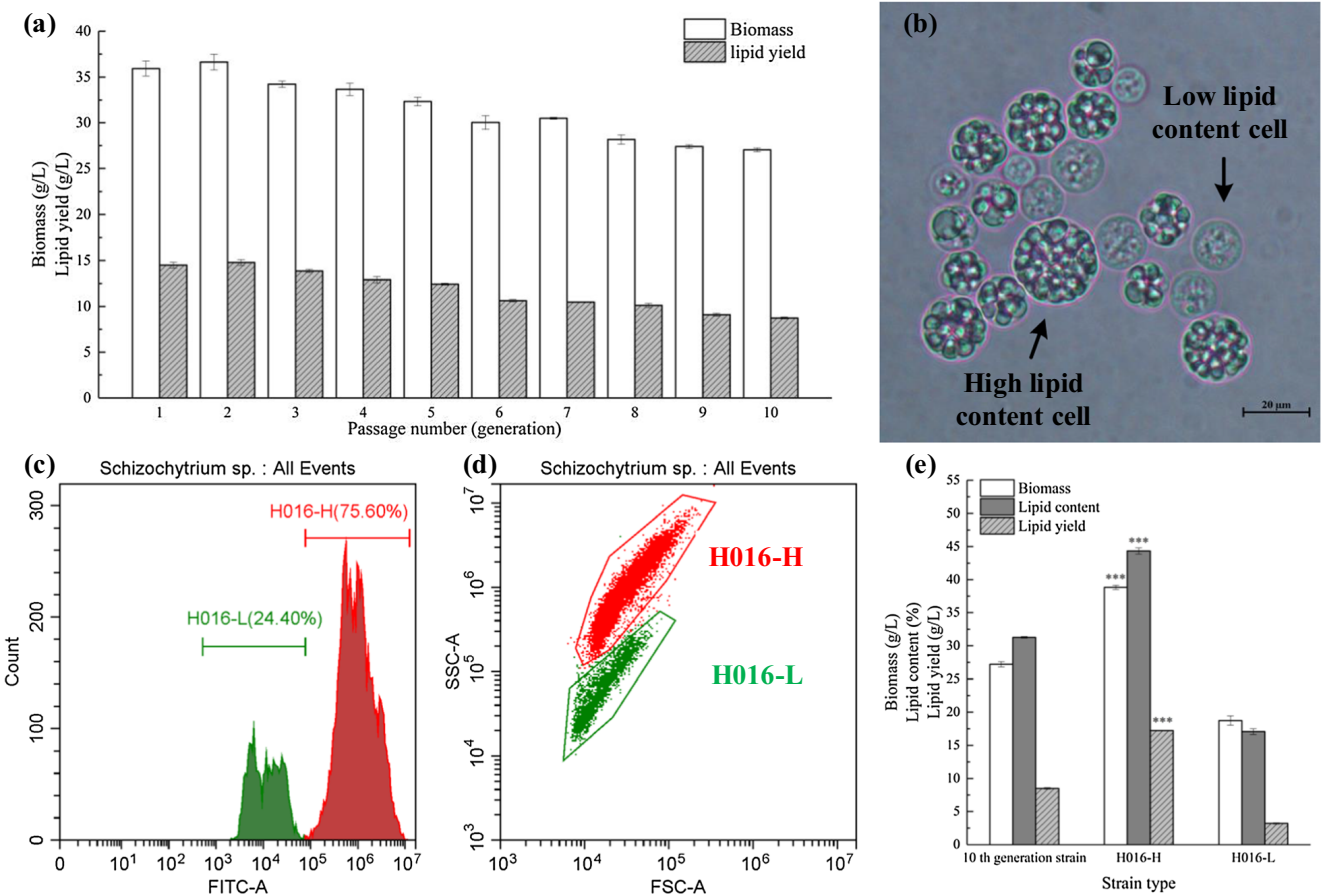
Comparative analysis of the fermentation performances and morphological characteristics of *Schizochytrium* sp. H016 after consecutive subculturing. **a** Biomasses and lipid yields of *Schizochytrium* sp. H016 during subculture. **b** Observation of the 10th generation strain under optical microscopy. **c** Flow cytometry analysis. The histogram shows the distribution of Nile red fluorescence intensity. FITC (x-axis) reflects the fluorescence intensity, and Count (y-axis) is the number of cells. **d** Dot blots of *Schizochytrium* sp. H016 subpopulations. The red dots are H016-H, and the green dots are H016-L. **e** Biomass, lipid content and lipid yield of the 10th generation strain and sorted strains

*Schizochytrium* sp. H016 was used as the initial strain to monitor the fluctuation of biomass and lipid yield during consecutive subculturing. As shown in Fig. 1a, the biomass and lipid yield of *Schizochytrium* sp. H016 decreased gradually during consecutive passage and culture processes. The biomass and lipid yield of the 10th generation strain reached 27.03 and 8.74 g/L, respectively, which were 24.77% and 39.68% lower than those of the initial strain. Subsequently, the morphological characteristics of the cells cultured for 144 h in fermentation medium were observed under optical microscopy. Interestingly, significant cellular heterogeneity was present in the fermentation broth of the 10th generation strain (Fig. 1b). One group of cells presented large particle sizes with dense lipid bodies, while another group showed smaller particle size without obvious lipid bodies. Therefore, we speculated that the content of intracellular lipid droplets has an important relationship with lipid production and that the existence of degenerated lipid droplet cells would reduce the total lipid yield of the overall fermentation system. FACS was used to separate the two cell subpopulations to test this hypothesis. Intriguingly, the heterogeneous cells differentiated into two subpopulations after Nile red staining and flow cytometry analysis (Fig. 1c, 1d). Of these cells, 24.40% presented low Nile red fluorescence intensity, whereas 75.60% had strong fluorescence intensity. Herein, these two cell subgroups were sorted as the representatives of the homogeneous populations with high and low lipid contents (i.e., H016-H and H016-L) for the further study of the physiological and fermentation characteristics of *Schizochytrium* sp.

Flow cytometry can not only separate subpopulations with different fluorescence intensities, but also directly reflect cell size and intracellular complexity through the optical FSC and SSC signals of single cell passing through channels [18]. The higher FSC and SSC intensities observed in H016-H than in H016-L suggested that H016-H had a larger cell size and more complicated intracellular structure than H016-L (Fig. 1d). Furthermore, the fermentation results showed that lipid production of H016-H (17.22 g/L) was significantly enhanced compared with that of the 10th generation strain (8.50 g/L) and that the lipid yield had increased by 102.59% (Fig. 1e). Moreover, a significant difference was observed between H016-H and H016-L in terms of biomass, lipid content and lipid yield. These data suggested that the presence of cell subpopulations with degenerated lipid droplets significantly decreased the overall lipid yield of the fermentation system and indicated that FACS is an effective separation method for obtaining homogenous cells with high lipid contents.

### Comparison of the morphological characteristics and lipid distribution of the two cell subpopulations

**Fig. 2:**
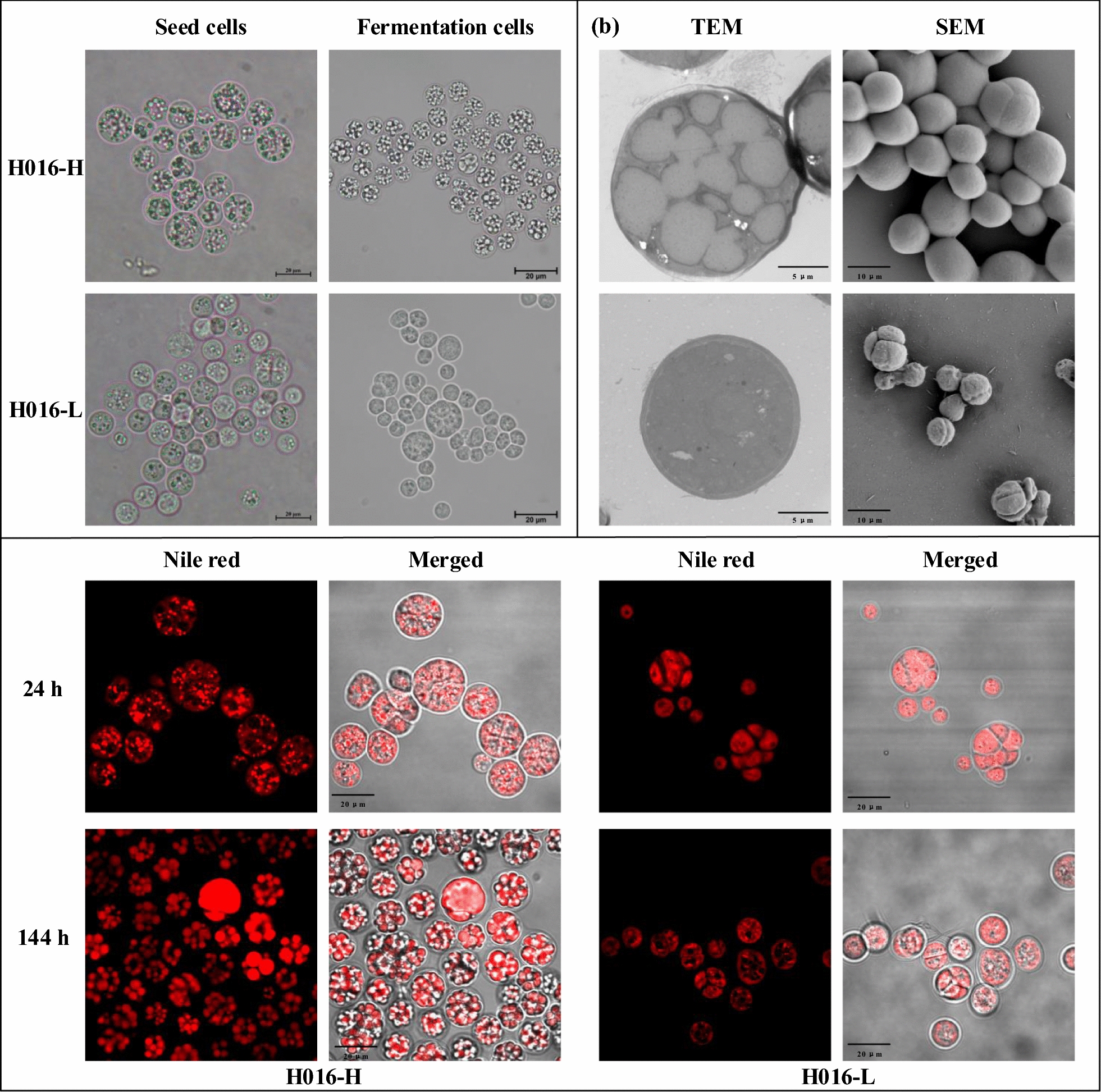
Microscopic morphology of H016-H and H016-L. Optical microscopy, SEM, TEM, and LSCM observations of cell morphology

Elucidating changes in microbial cell morphology is crucial for investigating the dynamics of lipid accumulation. In the fermentation culture, H016-H was found to have inherited the strong lipid synthesis capability of the seed cells, and its intracellular lipid droplets were greater in number and fuller in morphology than those of H016-L (Fig. 2a). This phenomenon was also verified via transmission electron microscopy (TEM). Interestingly, the scanning electron microscopy (SEM) images revealed obvious differences in cell division between the two subpopulations (Fig. 2b). Most cells in H016-L had small sizes and wizened surfaces at the dividing stage, indicating that H016-L possessed strong cell division capability and low intracellular contents. By contrast, most H016-H cells were separated vegetative cells with large sizes and abundant contents. Furthermore, propidium iodide (PI) was used to examine the cell proliferation capability of the two subpopulations at the logarithmic growth period. The fluorescent probe PI specifically binds intracellular DNA to produce fluorescence at an excitation wavelength of 535 nm, thereby reflecting DNA replication capability [19]. The considerably higher fluorescence intensity in H016-L than in H016-H indicated that H016-L had stronger cell proliferation activity than H016-H (Additional file 1: Figure S1).

Cell samples that had been cultivated for 24 and 144 h were stained with Nile red and observed under laser scanning confocal microscopy (LSCM) to determine the distribution of neutral lipids in cells. Neutral lipids, as a relatively stable substance, are stored in abundance in the cytosolic lipid bodies of oleaginous microorganisms [20]. In H016-H, the lipid bodies gradually enlarged, and fluorescence intensity increased with the prolongation of the fermentation time. By contrast, no identifiable subcellular structures were present in H016-L. The fluorescence intensity of H016-L decreased with fermentation time, and only the cell surface exhibited fluorescent signals (Fig. 2c). As such, the low lipid content in H016-L was due to the degradation of intracellular lipid droplets. Notably, when the cell subpopulations obtained after sorting were recultivated, no cell heterogeneity was observed. This phenotype strongly suggested that homogeneous cells can be obtained through single-cell sorting.

### Characterization of the fermentation behaviors of H016-H and H016-L

**Fig. 3:**
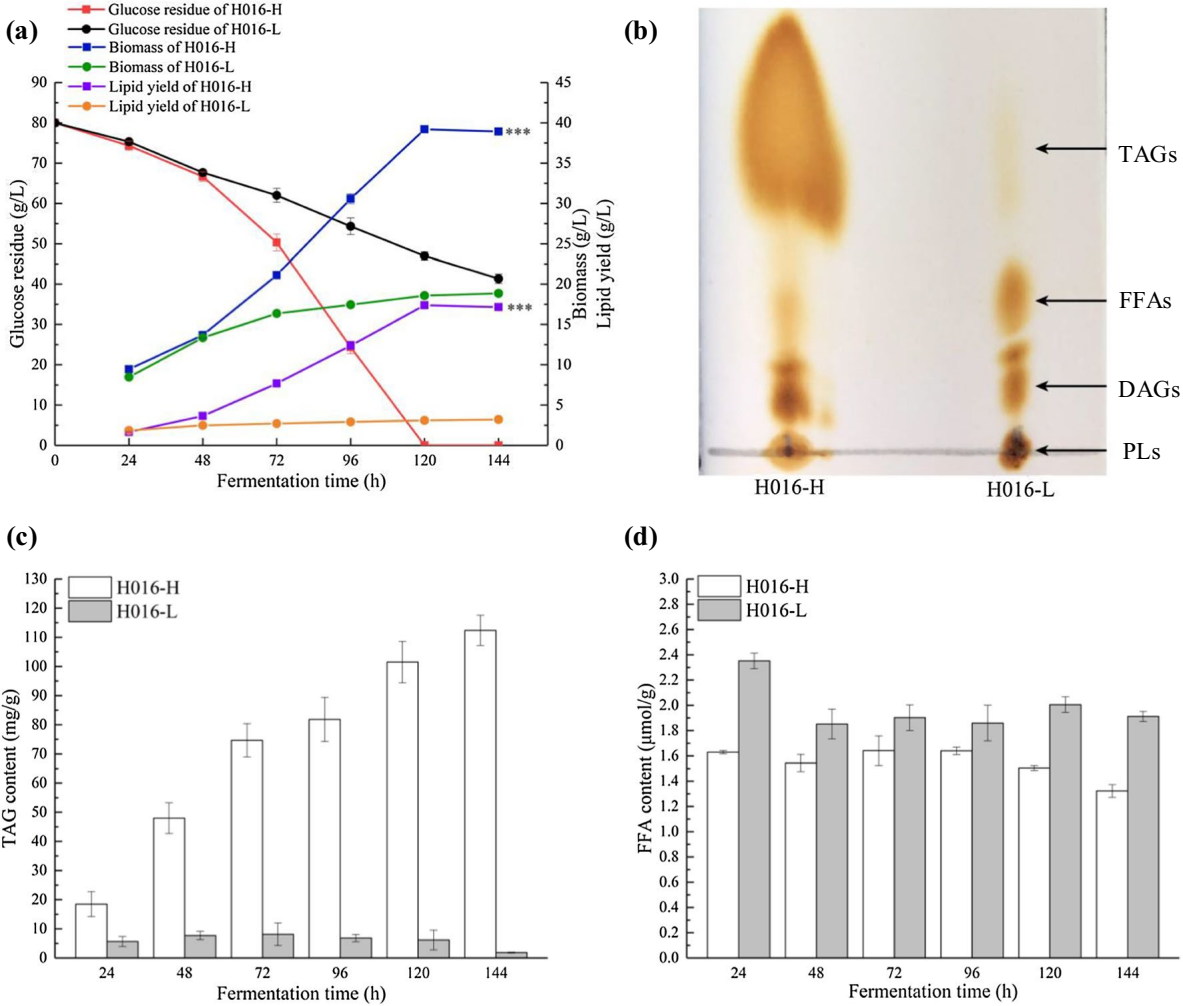
Growth kinetics analyses and fatty acid composition of H016-H and H016-L. **a** Growth curves based on glucose residues, biomasses, and lipid yields. **b** TLC analysis of extracted lipids from H016-H (left lane) and H016-L (right lane). **c** TAG concentration of H016-H and H016-L during fermentation. **d** FFA concentration of H016-H and H016-L during fermentation

The fermentation characteristics of H016-H and H016-L during cultivation were compared. In the H016-H culture, glucose was completely exhausted at 120 h, and biomass and lipid yield reached the highest values of 39.21 and 17.38 g/L, respectively (Fig. 3a). Subsequently, biomass and lipid yield declined slightly as the cells shifted to the consumption of accumulated fatty acids. However, the glucose utilization capacity of H016-L decreased significantly, and 41.33 g/L glucose remained in the medium until the end of fermentation. The carbon source consumed by *Schizochytrium* sp. is mainly used for the synthesis of fatty acids, which are further converted into various lipids for storage in lipid droplets. The poor utilization of glucose by H016-L accounted for the low biomass and lipid yield. H016-H and H016-L had the final biomasses of 37.93 and 18.85 g/L, respectively, and the lipid yields of 17.16 and 3.21 g/L, respectively.

Microbial lipids are mainly composed of TAGs, diglycerides (DAGs), free fatty acid (FFAs), and a variety of polar lipids (PLs). TAGs account for more than 80% of the lipid mass in marine oleaginous microorganisms [21]. In *Schizochytrium* sp., fatty acids are synthesized and released in the form of FFAs and subsequently incorporated into TAGs as storage lipids or into PLs as membrane lipids [22]. The total lipid compositions of H016-H and H016-L were evaluated by using thin-layer chromatography (TLC). The chromatogram is presented in Fig. 3b. In contrast to that in H016-L, the TAG content accounted for the majority of the lipid components in H016-H. The lipids in H016-L mainly consisted of FFAs, DAGs, and PLs. Furthermore, the TAG synthesis capability of the two cell subpopulations was quantitatively evaluated. The time-series results showed that the TAG concentration in H016-H increased gradually and reached the maximum value (112.38 mg/g) at 144 h (Fig. 3c). However, no obvious accumulation of TAG was observed in H016-L, and the TAG content decreased in the later stage of fermentation. In addition, FFA content was higher in H016-L than in H016-H (Fig. 3c). Hence, these finding indicated that the low lipid yield of H016-L was due to the degeneration of TAG synthesis capability and the gradual decomposition of the synthesized TAGs into FFAs.

Fatty acid composition was subsequently evaluated via gas chromatography–mass spectrometry (GC–MS) analysis, and the fatty acids were found to consist mainly of C14:0, C16:0, DPA, and DHA (Table 1). In H016-H, the contents of DPA and DHA remained stable, whereas those of C14:0 and C16:0 showed the opposite trend because C14:0 was gradually consumed and converted into C16:0. At the end of fermentation, the DHA content in H016-H reached 57.24%. This result reflected the strong capability of the isolated single cell subpopulation for DHA synthesis. The fatty acid composition of H016-L was consistent with that of H016-H. However, the DHA content of H016-L decreased with fermentation time likely because it was gradually consumed. At the end of fermentation, only 39.87% of DHA remained in H016-L. This value was 30.35% lower than the remaining DHA content of H016-H.

**Table 1:** Comparison of the fatty acid profile between H016-H and H016-L

Fermentation time (h)	Fatty acids profile of H016-H (%)				Fatty acids profile of H016-L (%)			
	C14:0	C16:0	DPA	DHA	C14:0	C16:0	DPA	DHA
24	8.51 ± 0.63	13.43 ± 0.56	10.21 ± 0.21	54.21 ± 1.00	10.34 ± 1.02	16.51 ± 0.91	8.90 ± 0.69	50.52 ± 1.77
48	10.27 ± 1.44	12.37 ± 1.35	10.30 ± 1.12	53.13 ± 0.42	9.38 ± 0.73	19.17 ± 1.55	9.64 ± 0.14	50.05 ± 1.12
72	8.90 ± 1.05	12.27 ± 1.04	10.52 ± 0.81	57.12 ± 0.23	6.66 ± 0.03	20.66 ± 0.57	9.38 ± 0.37	46.41 ± 1.19
96	5.03 ± 0.59	17.04 ± 1.44	11.71 ± 0.50	58.18 ± 0.41	5.86 ± 0.61	24.45 ± 1.68	10.15 ± 0.09	43.52 ± 0.51
120	3.59 ± 0.42	18.64 ± 0.86	13.00 ± 0.50	56.73 ± 0.71	5.89 ± 0.71	26.94 ± 0.54	9.84 ± 0.07	41.31 ± 0.89
144	3.40 ± 0.03	17.96 ± 0.48	13.09 ± 0.45	57.24 ± 1.10	5.84 ± 0.32	30.38 ± 0.91	9.61 ± 0.37	39.87 ± 1.00

### Transcriptome analysis of H016-H and H016-L during fermentation

**Fig. 4:**
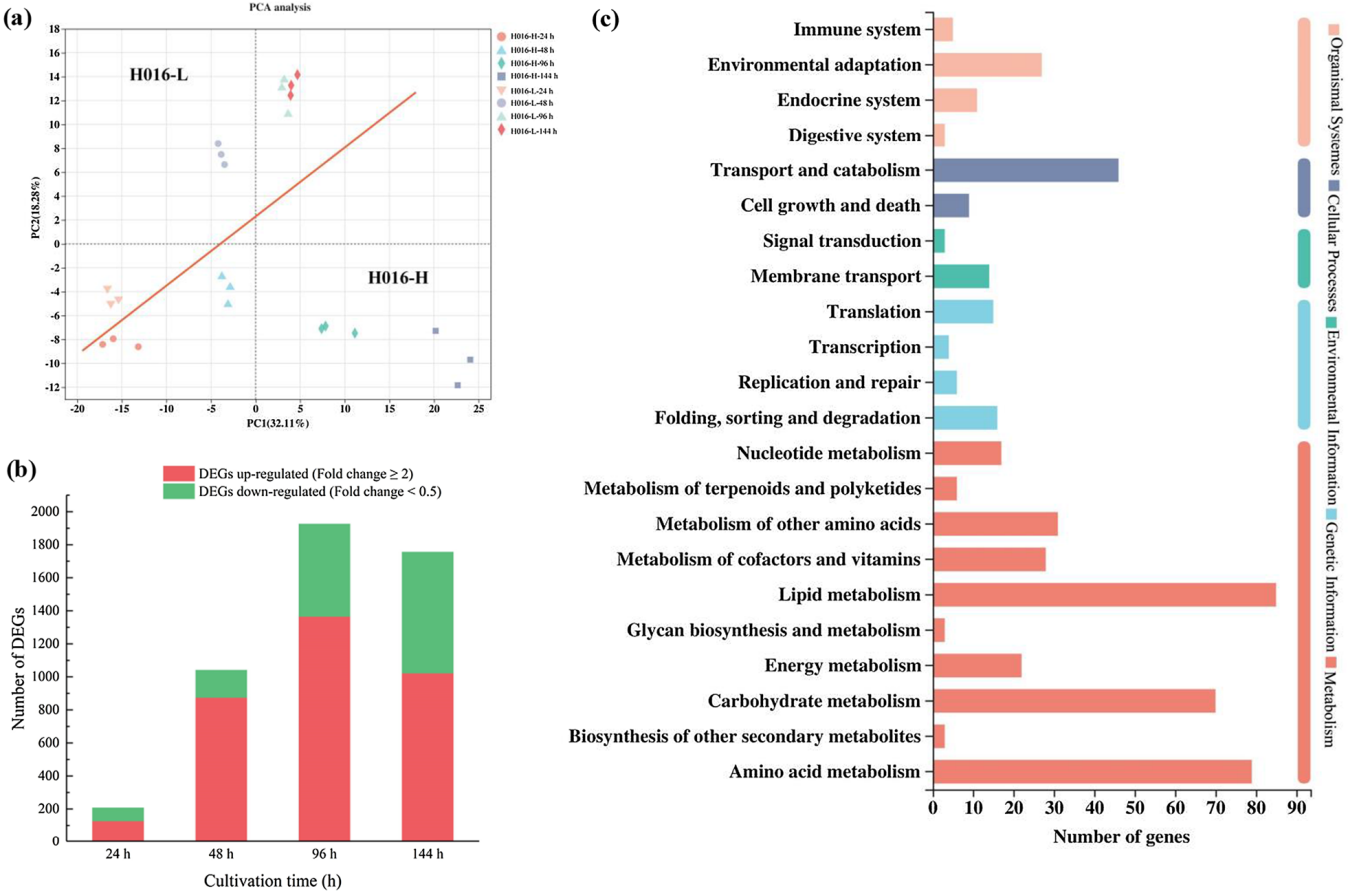
Overview of transcriptome analysis during the fermentation of the two cell subpopulations. **a** PCA plot shows the clustering between transcriptome samples. **b** DEG number of upregulated and downregulated genes in H016-H and H016-L at the four fermentation stages. **c** KEGG pathway enrichment analysis of all DEGs

Theoretical and experimental studies have indicated that gene expression noise can contribute to cell-to-cell phenotypic heterogeneity between subpopulations [23, 24]. The global transcriptome was used to analyze differential transcriptional regulation between H016-H and H016-L to analyze the molecular mechanisms underlying the phenotypic heterogeneity of *Schizochytrium* sp. adequately. Samples were collected at 24, 48, 96, and 144 h of the fermentation process. Principal component analysis (PCA) was performed on all of the expressed genes in the samples on the basis of the fragments per kilobase of transcript per million fragments (FPKM). The two subpopulations showed significant differences with time (Fig. 4a). The two groups of samples clustered together at 24 h. However, the distance between clusters increased at 48, 96, and 144 h, indicating that gene expression noise was gradually amplified during fermentation. A total of 19,796 transcripts were detected in the samples, and genes with *P*-adjust < 0.05 & |log_2_FC|≥ 1 were selected as differentially expressed genes (DEGs) for further analysis. By using the H016-L sample taken at the same time point as a control, the H016-H sample was found to have 126, 873, 1364, and 1021 genes upregulated and 82, 169, 563, and 736 genes downregulated at 24, 48, 96, and 144 h, respectively (Fig. 4b). The number of DEGs was lowest at 24 h (208) but gradually increased since then, indicating that the metabolic pathways in the two cell subpopulations differed significantly.

All DEGs were mapped by using the Kyoto Encyclopedia of Genes and Genomes (KEGG) to elucidate the key genes associated with phenotypic heterogeneity. As expected, most of the DEGs were enriched in the lipid metabolism pathway (Fig. 4c). This result was consistent with the observed phenotypes, specifically, the different lipid accumulation capabilities, of the two cell subpopulations. Notably, the DEGs involved in amino acid metabolism were highly enriched. The amino acid metabolic pathway provides energy and intermediate metabolites for lipid synthesis and plays a crucial role in cell cycle, transmembrane active transport, and cell signaling [25]. We also noticed that the DEGs were also significantly enriched in cell cycle pathways, including cell growth and death, DNA replication and repair, and nucleotide metabolism. The DEGs in these metabolic pathways provided additional evidence for the different cell proliferation phenomena observed in the two cell subpopulations.

### Differential regulation of genes determining the cell cycle and cell division

**Fig. 5:**
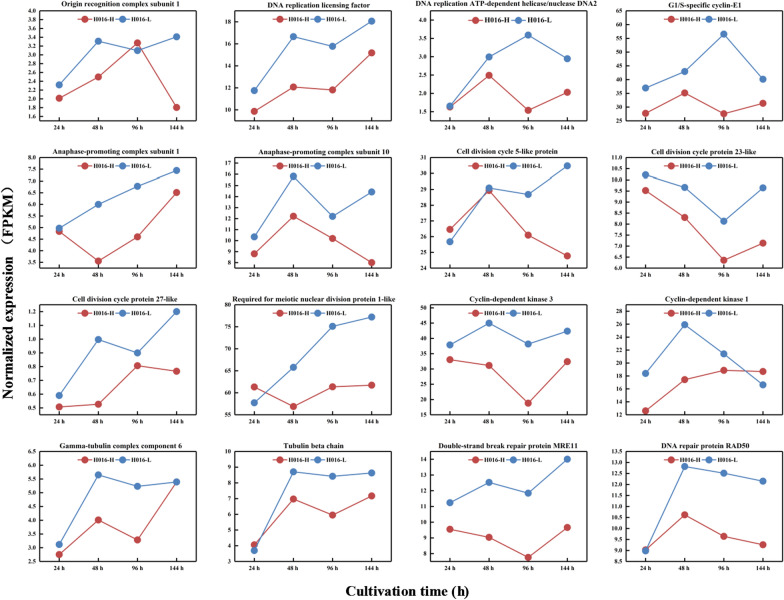
Differential expression of representative genes involved in various aspects of the cell cycle, including DNA replication, cell division, tubulin, and cell cycle regulation

Given that cell morphology was differed throughout the cell cycle, the expression profiles of key genes involved in DNA replication, cell division, tubulin, and cell cycle regulation were analyzed. Clustering heat map analysis on the set of genes annotated with the term “cell cycle” by Gene Ontology (GO) revealed that the overall expression of the gene family of H016-L was higher than that of H016-H (Additional file 1: Figure S2). In addition, genes related to cell cycle control were significantly upregulated in H016-L (Fig. 5). The expression levels of the DNA replication licensing factor, the DNA replication ATP-dependent helicase/nuclease encoding gene, and the origin recognition complex subunit 1 encoding gene were upregulated in H016-L and were involved in the initiation and regulation of DNA replication processes [26, 27]. The expression of genes encoding the components of G1/S-specific cyclin-E, anaphase-promoting complex subunit 1, anaphase-promoting complex subunit 10, cyclin-dependent kinase 1, and cyclin-dependent kinase 3 were also upregulated in H016-L relative to in H016-H. These genes are thought to play an important role in facilitating the cell cycle from the G1 phase to the S phase [28]. Eukaryotic cell division is a complex process that relies on a complex of multiple cell division proteins [29]. The genes encoding cell division cycle 5-like protein (CDC5L) and cell division cycle 23-like protein and required for meiotic nuclear division protein 1-like exhibited high transcriptional activity in H016-L. The CDC5L protein has been proven to regulate the G2/M transition of the mitotic cell cycle [30]. Microtubules are core components of the eukaryotic cytoskeleton with essential roles in cell division, shaping, motility and intracellular transport [31]. The expression of genes encoding the components of tubulin, such as tubulin complex component 6 and tubulin beta chain, was also higher in H016-L than in H016-H. Furthermore, the genes encoding the double-strand break repair protein MRE11 and the DNA repair protein RAD50 showed significantly higher expression levels in H016-L than in H016-H and have been implicated in double‐strand break repair in eukaryotic cells [32, 33].

### Global transcriptional analysis of central carbon metabolism and lipid-related pathways

**Fig. 6:**
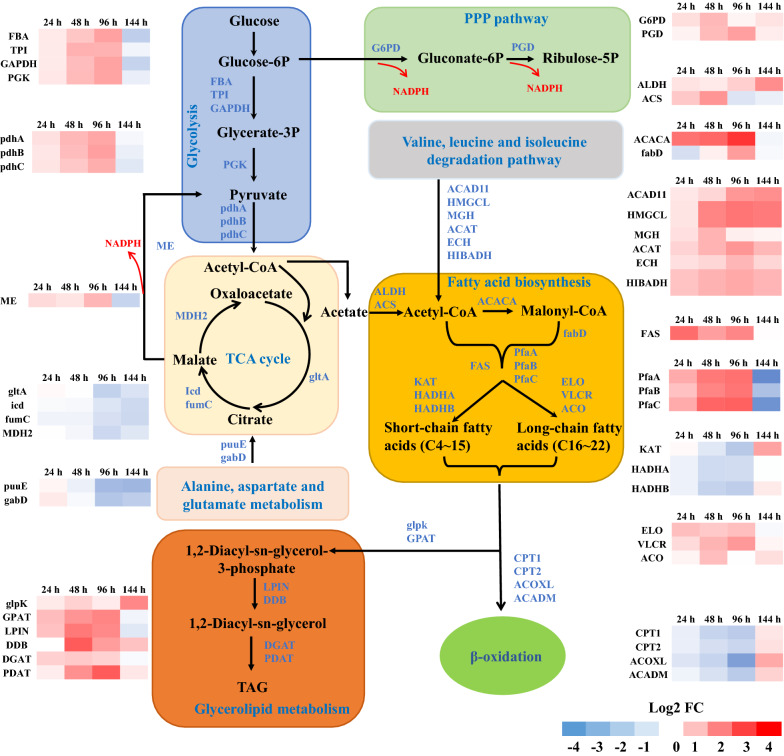
Overview of the transcriptional reprogramming of the central carbon metabolic and lipid-related pathways in H016-H and H016-L at 24, 48, 96, and 144 h. The heatmaps show transcriptional differences in log_2_ (fold change) in H016-H relative to those in H016-L

In oleaginous microorganisms, central carbon metabolism is the main source of acetyl-CoA and NADPH, which are the precursor and reducing agent of lipid synthesis, respectively. At 24, 48, and 96 h, the expression levels of genes encoding fructose-bisphosphate aldolase (*FBA*), triosephosphate isomerase (*TPI*), glyceraldehyde-3-phosphate dehydrogenase (*GAPDH*), and phosphoglycerate kinase (*PGK*), which participate in the glycolysis pathway, in H016-H were upregulated relative to those in H016-L. Subsequently, pyruvate produced by glycolysis was transported into the mitochondria and further converted into acetyl-CoA to participate in the TCA cycle. The pyruvate dehydrogenase genes *pdhA*, *pdhB*, and *pdhC* that catalyze the conversion of pyruvate into acetyl-CoA in this process were significantly upregulated in H016-H at 24, 48 and 96 h. In addition, the results of DEG analysis revealed that throughout the fermentation process, the expression of key genes in the valine, leucine, and isoleucine degradation pathway in H016-H were significantly upregulated compared with those in H016-L. These key genes included those encoding acyl-CoA dehydrogenase family member 11 (*ACAD11*), hydroxymethylglutaryl-CoA lyase (*HMGCL*), methylglutaconyl-CoA hydratase (*MGH*), acetyl-CoA acetyltransferase (*ACAT*), short-chain-enoyl-CoA hydratase (*ECH*), and 3-hydroxyisobutyrate dehydrogenase (*HIBADH*). The significant enhancement of the valine, leucine, and isoleucine degradation pathway could increase the production of acetyl-CoA, which is a precursor for fatty acid synthesis (Fig. 6).

Acetyl-CoA is the substrate for the further synthesis of the major metabolite fatty acids. In terms of fatty acid composition, H016-H showed a higher DHA content than H016-L (Table 1). At 24, 48, and 96 h, the genes involved in the fatty acid synthase (FAS) pathway and polyketide synthase (PKS, *PfaA*, *PfaB* and *PfaC* subunits) pathway were upregulated in H016-H compared with those in H016-L. Interestingly, significant differences were observed in the expression levels of the genes involved in the synthesis of short-chain and long-chain fatty acids between H016-H and H016-L. Compared with those in H016-L, the genes in H016-H involved in catalyzing the synthesis of long-chain fatty acids, such as elongation of very long chain fatty acids protein 5 (*ELO*) and very-long-chain 3-oxoacyl-CoA reductase (*VLCR*), were significantly upregulated at 24, 48, and 96 h. Simultaneously, short-chain fatty acid synthesis genes, such as 3-ketoacyl-CoA thiolase (*KAT*), trifunctional enzyme subunit alpha (*HADHA*), and acetyl-CoA acetyltransferase (*HADHB*) in H016-H were significantly downregulated compared with those in H016-L. This situation may be highly favorable for the synthesis of long-chain PUFAs, such as DPA and DHA. In addition, NADPH is known to be a key cofactor required for fatty acid synthesis and desaturation and is mainly produced by the enzymatic reaction of malic enzyme (*ME*), glucose-6-phosphate dehydrogenase (*G6PD*) and 6-phosphogluconate dehydrogenase (*PGD*) [34]. The higher expression levels of these three key genes in H016-H than in H016-L facilitated the production of additional NADPH for the synthesis of PUFAs. The genes that participate in the glycerolipid production pathway, such as glycerol kinase (*glpK*), phosphatidate phosphatase (*LPIN*), PA-phosphatase related-family protein DDB (*DDB*), diacylglycerol acyltransferase (*PDAT*), diacylglycerol O-acyltransferase (*DGAT*), and phospholipid acyltransferase 2 (*GPAT*), were upregulated in H016-H relative to in H016-L. By contrast, lipid degradation pathway-related genes such as carnitine O-palmitoyltransferase (*CPT1* and *CPT2*), acyl-coenzyme A oxidase-like protein (*ACOXL*), and medium-chain specific acyl-CoA dehydrogenase (*ACADM*) were downregulated in H016-H. Notably, at 144 h, genes in glucolysis and fatty acid biosynthesis showed higher activity in H016-L than in H016-H because in H016-H, glucose was depleted at 120 h and the metabolic direction was switched to β-oxidation for the decomposition of synthesized lipids to maintain cell metabolism and energy supply.

## Discussion

In the present study, the fluorescence intensity of Nile red was innovatively used as a screening marker for the FACS sorting of phenotypically heterogeneous cell subpopulations of *Schizochytrium* sp. H016, and transcriptome analysis was performed on the selected cell subpopulations. The findings of this work are important because they provide a novel breeding strategy and new insight into the phenotypic heterogeneity of *Schizochytrium* sp. In previous works, the interference of cell heterogeneity on study results was ignored. To our knowledge, this is the first study on the phenotypic heterogeneity of *Schizochytrium* sp. Population heterogeneity is a common phenomenon in microbial populations. Researchers have long noticed a decline in metabolite production after the long-term culture of strains and attributed this phenomenon to strain degradation [35]. However, this explanation is not comprehensive because strains with excellent phenotypes can usually be isolated from existing cultures. In fact, phenotypic heterogeneity is the essential reason for the occurrence of different populations of microorganisms. In response to the rapidly changing environment, some microorganisms can produce populations with multiple phenotypes in terms of metabolism, gene expression and growth to achieve the ultimate goal of survival in a competitive environment [36]. Nevertheless, microbial heterogeneity can affect the robustness of biological processes and the precise control of fermentation processes; this phenomenon has a variety of effects on productivity [37]. Therefore, the reduction of phenotypic heterogeneity and the acquisition of homogeneous populations have attracted considerable attention [37].

A typical feature of population heterogeneity is phenotypic differences at the single-cell level that can be observed under microscopy. This study confirmed the existence of two different subpopulations with significantly different lipid droplet contents after consecutive subculture (Fig. 1b). This result was also confirmed via flow cytometry analyses (Fig. 1c, d). The multi-peaked distribution behavior of fluorescence intensity is considered to be the most distinctive feature of population phenotypic heterogeneity. In this study, a novel efficient procedure was successfully applied to identify and isolate *Schizochytrium* sp. through FACS sorting. The long-passaged cells diverged into two groups on the dot plot, and gates were established in accordance with this feature to isolate the populations with different lipid contents. Our result was in agreement with previous findings showing that cell-to-cell variations in protein and metabolite concentrations are naturally inherent and that high- and low-performing variants exist in all cultures [38]. The sorted subpopulations were recultured and monitored for fermentation performance and cell morphology to clarify that the different cell subpopulations of *Schizochytrium* sp. H016 are not the result of different cell cycle stages (Figs. 1e, 2). The reculture results refuted the interference of the cell cycle on cell morphology, and the two cell subpopulations showed good homogeneity after recultivation, indicating that FACS sorting is a novel and effective breeding method.

Phenotypic heterogeneity may be beneficial to the division of the labor strategies of microbial communities to cope with complex tasks in specific environments [39]. In this study, we observed that the subpopulation with low lipid contents showed high cell division activity. *Schizochytrium* sp. can divide in a variety of ways that may be a stress response for adaptation to the growth environment [40]. However, very little is known about the relationship between lipid accumulation and cell cycle regulation in *Schizochytrium* sp. Generally, proliferating cells switch to metabolism with low efficiency [41]. The cells of *Aurantiochytrium acetophilum* HS-399, a closely related species of *Schizochytrium* sp., can be distinguished into rapidly dividing sporogenous cells and vegetative cells that accumulate large amounts of lipids. Similarly, previous reports have shown that in *Corynebacterium glutamicum*, slow-growing producers can overgrow by rapidly splitting non-producers, severely reducing the total production of L-valine [42]. Although a homogenized subpopulation with high lipid content was obtained through FACS sorting in this study, the molecular mechanism of the phenotypic heterogeneity of *Schizochytrium* sp. remains unclear.

Monitoring gene expression differences in cell subpopulations is a prerequisite for the further study of phenotypic heterogeneity [43]. Moreover, in *Schizochytrium* sp., the molecular mechanisms underlying the cell cycle are poorly understood. Finding key transcription factors or signal networks to modify the dynamics of the entire metabolic pathway is a necessary measure to achieve a balance between growth and lipid accumulation in *Schizochytrium* sp. In this study, RNA sequencing technology was utilized to detect the transcriptional differences between H016-H and H016-L at four different fermentation stages. Although the differences in biomass, lipid production, and gene expression between these two cell subpopulations at 24 h of fermentation were not significant, these differences gradually increased with the extension of culture time (Figs. 3a, 4a, b). KEGG analysis revealed that consistent with the phenotypic differences we observed, the DEGs were mainly involved in the cell cycle and lipid metabolism.

During the cell cycle, the genetic material of the replicating cell is distributed to the daughter cells at the end of division. The cell cycle is commonly divided into the dormant phase (G0), the early DNA synthesis phase (G1), the DNA synthesis phase (S), the late DNA synthesis phase (G2), and the division period (M). The entire cycle can be expressed as G0 → G1 → S → G2 → M. DNA replication in eukaryotic cells is initiated through the action of multiprotein complexes that recognize replication start sites in the chromosome (requiring the origin recognition complex) and facilitate duplex DNA melting within these regions (requiring DNA helicase) [29]. Then, G1/S-specific cyclin-E1 controls the G1/S transition [44]. The anaphase-promoting complex is a multi-subunit protein that regulates progression through the mitotic phase of the cell cycle and controls entry into the S phase [45]. Cyclin-dependent kinases also play an important role in the regulation of the G1/S cell cycle transformation, leading to increased cell proliferation and transformation [46, 47]. Finally, cells enter the M phase under the synergistic action of a series of cell division proteins. In this study, the significant enhancement in the transcriptional activity of cell cycle-regulating genes in H016-L compared with that in H016-H resulted in the higher cell division activity in H016-L.

Another typical difference between the two cell subpopulations was lipid metabolism. H016-L exhibited low glucose utilization and low TAG content under the conditions of adequate nutrition. Meanwhile, the expression of key genes in the glycolytic pathway of H016-L were also downregulated. Notably, the expression of key genes in the TCA cycle and metabolic pathways of alanine, aspartate, and glutamate, which provide precursors for the TCA cycle, in H016-H were significantly downregulated relative to those in H016-L; this situation may be favorable for the synthesis of cytoplasmic acetyl-CoA [48, 49]. The enhanced expression of the key enzyme genes encoding aldehyde dehydrogenase (*ALDH*) and acetyl-coA synthetase (*ACS*), which catalyze the conversion of acetate into acetyl-CoA and the weakening of the TCA cycle, is more conducive to the accumulation of acetyl-CoA in H016-H than in H016-L. Acetyl-CoA and malonyl-CoA are precursors for fatty acid synthesis [50]. Previous studies have demonstrated that the overexpression of the *ACS* gene can increase substrate supply and enhance the synthesis of fatty acids and lipids in *Schizochytrium* sp. ATCC 20,888 [51]. However, the specific pathway for the conversion of acetyl-CoA into long-chain PUFAs in *Schizochytrium* sp. remains unclear [52]. Existing theories state that *Schizochytrium* sp. may synthesize fatty acids via the FAS and PKS pathways [53, 54]. In *Schizochytrium* sp. S056, FAS genes have been reported to be upregulated when glycerol was used as the carbon resource and the DHA content was elevated [48]. By contrast, *Schizochytrium* sp. with high DHA content obtained through domestication in a low-temperature and high-salt environment showed strong PKS gene expression activity [55]. Compared with those in H016-L, the FAS and PKS genes in H016-H showed significant upregulation at 24, 48, and 96 h, indicating a high capacity for fatty acid synthesis. Fatty acids synthesized by marine oleaginous microorganisms are commonly released in the form of FFAs and subsequently incorporated into glycerolipids. As illustrated in Fig. 3b, fatty acids in H016-H were mainly converted into TAG and accumulate in lipid droplets, whereas FFAs were mainly present in H016-L. This difference could be explained by the differences in the expression of genes related to the glycerolipid metabolism pathway and β-oxidation between H016-H and H016-L.

## Conclusions

*Schizochytrium* sp. H016 subjected to long-term subculture was sorted by FACS, and the two obtained cell subpopulations showed opposite morphologies and lipid synthesis capacities. This work is the first piece of evidence for the phenotypically heterogeneous populations of *Schizochytrium* sp. The presence of the subpopulation with degraded lipid droplets resulted in a dramatic decrease in total biomass and lipid yield that seriously affected the large-scale fermentation production of DHA. Hence, a novel efficient screening method was applied to isolate the strain with high lipid content. On this basis, the phenotypic and fermentation characteristics of phenotypically heterogeneous subgroups were compared, and significant differences were found in lipid accumulation and the cell cycle. The results of transcriptome analysis were also consistent with and support the above results. In summary, this study provided new insights into the phenotypic heterogeneity of *Schizochytrium* sp. and demonstrated that FACS can be used as an effective strategy for the breeding of homogeneous strains with high lipid production.

## Methods

### Microorganism isolation and culture conditions

*Schizochytrium* sp. H016 (China Center for Type Culture Collection [CCTCC] M 2,021,260) was isolated from seawater from Qingdao, China, and stored in the CCTCC [56]. *Schizochytrium* sp. H016 was stored in 40% (v/v) glycerol at − 80 °C and activated and preserved once a month. A tube of the strain with good lipid biosynthesis capability was selected as the original strain. The strain preserved in the glycerin tube was inoculated into the seed culture medium and cultivated for 48 h. The seed medium (4%, v/v) was then transferred into the fermentation medium and cultured at 25 °C and the constant speed of 200 rpm for 144 h. The seed culture medium consisted of 50 g/L glucose, 2 g/L peptone, 5 g/L yeast extract, 0.5 g/L MgSO_4_·7H_2_O, 1.5 g/L KH_2_PO_4_, and 20 g/L seawater crystals. The fermentation culture medium consisted of 80 g/L glucose, 15 g/L yeast extract, 1.5 g/L KH_2_PO_4_, 0.5 g/L MgSO_4_·7H_2_O, 3 g/L NaNO_3_, and 20 g/L seawater crystals. This strain was cultured continuously for 10 generations. Specifically, a new cycle was started once a month by inoculating the strain into fresh seed medium followed by preserving the seed inoculum again and evaluating the fermentation performance. The medium and culture conditions for continuous culture passage are the same as those described above.

### Flow cytometry analysis and isolation of different cell subsets

Nile red was dissolved in acetone to the concentration of 0.1 mg/mL and added to the cell culture at a ratio of 1:100 for 20 min in the dark. The samples were filtered through a 40 μm cell filter and analyzed and sorted with a MoFlo XDP Cell Sorter (Beckman Coulter, USA). The Nile red signal was detected at 525 nm (FITC channel). The dot plots of the Nile red fluorescence intensities of the cells were used to establish the gates for isolating the populations of strains with high and low lipid contents.

### Cell morphology and lipid droplet observation

Cells cultured for 48 h in seed medium and 144 h in fermentation medium were diluted tenfold and observed under an inverted fluorescence microscope (Ti-2, Nikon, Japan). Microbial cells were stained with Nile red and observed under a laser confocal microscope (FV1000, Olympus, Japan) at the excitation wavelength of 488 nm and emission wavelength of 505–550 nm to observe the distribution of lipid bodies [34]. The cell samples that had been cultured for 24, 72, 120, and144 h were collected, washed twice with 0.1 M phosphate buffer, and centrifuged at 1000 rpm for 5 min to observe cell division. The cells were fixed with 2.5% glutaraldehyde for 2 h at room temperature. The samples were washed three times with phosphate buffer (pH 7.4) and fixed for 2 h with 0.1 M phosphate buffer containing 1% osmium tetroxide solution. Then, the samples were dehydrated with 30%–100% ethanol. Finally, the samples were mounted on stubs for gold sputtering and then observed through SEM (SU8100, HITACHI, Japan). TEM was performed to study the differences in cell ultrastructures. The procedure for TEM was similar to that for SEM. Specifically, the samples collected at 144 h were treated with Epon 812 embedding medium after dehydration in a graded series of acetone and observed under a TEM (HT7700, HITACHI, Japan).

### Determination of biomass, glucose residue, lipid content, and fatty acid methyl esters

The cell samples were centrifuged at 8000 rpm for 10 min at 4 °C and washed three times with ultrapure water. Then, the samples were transferred to filter paper and dried in a vacuum oven at 60 °C until their weight remained constant. The glucose residue was analyzed by using a bioanalyzer (SBA-40D, China). The dried cell samples were crushed by using a pulverizer, and the lipids were extracted three times with n-hexane. Fatty acid composition in terms of fatty acid methyl esters was analyzed via GC–MS in accordance with our previous publications [48].

### TLC analysis of lipid composition and assay of TAG and FFA content

TLC analysis was performed in accordance with a previous method with some modifications [57]. The total lipid extracts dissolved in chloroform were separated through TLC (silica gel GF254, 25 mm × 75 mm, 0.25 mm thickness, Qingdao Haiyang, China) in a mixture of hexane/diethyl ether/acetic acid (80:20:2, v/v/v) until the layers separated. The plate was stained with iodine vapor at 37 °C for 10 min to separate TAG, DAG, FFAs, and PLs.

FFA content was determined by using a FFA content detection kit (BC0595, Solarbio, China). Data were analyzed in accordance with the instruction manual. The determination principle is that under weakly acidic conditions, FFAs react with copper salt to produce fatty acid copper salt, and a characteristic absorption peak appears at 550 nm. Therefore, content of FFAs has a linear relationship with the degree of color development. TAG content was determined with a TAG content detection kit (BC0590, Solarbio, China) in accordance with a previous study [58].

### Transcriptomics analysis

H016-H and H016-L samples were collected at 24, 48, 96 and 144 h of cultivation with three biological replications. TRIzol reagent (Invitrogen) was used to isolate the total RNA. Oligo (dT) magnetic beads were used to pair A-T bases with polyA to isolate mRNA from total RNA for transcriptome information analysis. Fragmentation buffer was added to break the mRNA randomly into small fragments of approximately 300 bp and then used for cDNA synthesis. Then, cDNA ends were repaired with end repair mix and ligated to Illumina sequencing adapters. Finally, 2 × 150 bp paired-end sequencing was performed on an Illumina Novaseq 6000 platform (Illumina, CA, USA). The original sequencing data were filtered to remove adapter sequences, low-quality reads, sequences with a high rate of uncertain base sequences, and sequences that were too short to obtain clean data. The clean data were compared with the reference genome to obtain the mapped data for subsequent analysis. FPKM was used to calculate the transcript expression levels. DESeq2 was used to identify DEGs. Genes with adjusted *P* value (*P*-adjust) < 0.05 and upregulated or downregulated fold change ≥ 2 were identified as DEGs. Finally, the DEGs were mapped to the KEGG database for enrichment analysis. A KEGG pathway was considered to be significantly enriched when *P*-adjust < 0.05.

## Supplementary Information

**Additional file 1: Figure S1.** Comparative analysis of fluorescence intensity of two cell subsets after PI staining. **Figure S2.** Heatmap showing the relative expression of DEGs related to cell cycle at each stage in H016-H and H016-L. **Figure S3.** Scanning electron microscopy observation of cell division status during fermentation process of two cell subpopulations. **Figure S4.** The volcano map of difference expression genes between H016-H and H016-L in fermentation process. **Figure S5.** Go classification of difference expression genes between H016-H and H016-L. **Figure S6.** KEGG classification of difference expression genes between H016-H and H016-L. **Figure S7.** Expression trends of genes in six clusters. The gray lines represent the expression levels of individual genes. The blue lines represent the average expression level of genes in the cluster. **Figure S8.** KEGG enrichment analysis of differentially expressed genes in two cell subpopulations during fermentation.

## Data Availability

All data generated or analyzed during this study are included in this published article and its additional files.

## Abbreviations

FACS: Fluorescence-activated cell sorting
SFAs: Saturated fatty acids
C16:0: Palmitic acid
C18:0: Stearic acid
PUFAs: Polyunsaturated fatty acids
EPA: Eicosapentaenoic acid
DHA: Docosahexaenoic acid
PKS: Polyketide synthase
FAS: Fatty acid synthetase
TAG: Triacylglycerol
TEM: Transmission electron microscopy
FAMEs: Fatty acid methyl esters
TLC: Thin-layer chromatography
CFW: Cell fresh weight
FFA: Free fatty acid
SSC: Side scatter
FSC: Forward scatter
DAG: Diglyceride
PLs: Polar lipids
