# Supplementary material for: New insights into phenotypic heterogeneity for the distinct lipid accumulation of Schizochytrium sp. H016
Source: Biotechnol Biofuels Bioprod. 2022 Mar 25;15:33. doi: 10.1186/s13068-022-02126-w (PMC8957170; doi:10.1186/s13068-022-02126-w)
Supplement: Supplementary file 1 — Additional file 1: Figure S1. Comparative analysis of fluorescence intensity of two cell subsets after PI staining. Figure S2. Heatmap showing the relative expression of DEGs related to cell cycle at each stage in H016-H and H016-L. Figure S3. Scanning electron microscopy observation of cell division status during fermentation process of two cell subpopulations. Figure S4. The volcano map of difference expression genes between H016-H and H016-L in fermentation process. Figure S5. Go classification of difference expression genes between H016-H and H016-L. Figure S6. KEGG classification of difference expression genes between H016-H and H016-L. Figure S7. Expression trends of genes in six clusters. The gray lines represent the expression levels of individual genes. The blue lines represent the average expression level of genes in the cluster. Figure S8. KEGG enrichment analysis of differentially expressed genes in two cell subpopulations during fermentation. [file 13068_2022_2126_MOESM1_ESM.docx]

**Additional Information**

**New insights into phenotypic heterogeneity for the distinct lipid accumulation of *Schizochytrium* sp. H016**

Zhendong Bao^1,2,3†^, Yuanmin Zhu^1,2,3†^, Yumei Feng^1,2,3^, Kai Zhang^1,2,3^, Meng Zhang^1,2,3^, Ruili Li^1^, Longjiang Yu^1,2,3*^

^1^ Institute of Resource Biology and Biotechnology, Department of Biotechnology, College of Life Science and Technology, Huazhong University of Science and Technology, Wuhan 430074, China;

^2^ Key Laboratory of Molecular Biophysics, Ministry of Education, Wuhan 430074, China;

^3^ Hubei Engineering Research Center for both Edible and Medicinal Resources, Wuhan 430074, China;

† These two authors contributed equally to this work.

*** Correspondence:**

LongJiang Yu, College of Life Science and Technology, Huazhong University of Science and Technology, No. 1037 Luoyu Road, Wuhan 430074, China. Tel.: +86 27 87792264; fax: +86 27 87792265

E-mail address: [yulongjiang@hust.edu.cn](mailto:yulongjiang@hust.edu.cn).


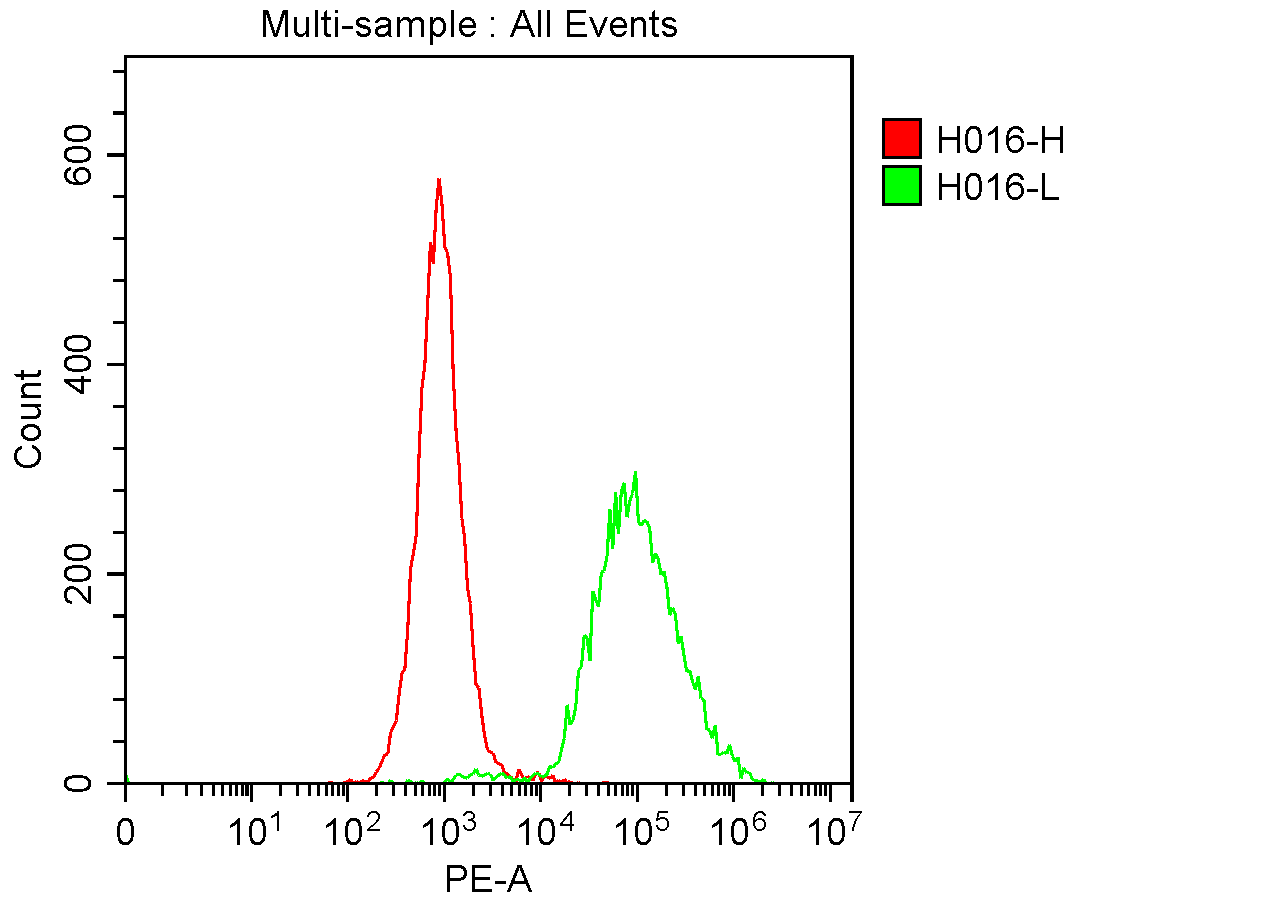


Figure S1. Comparative analysis of fluorescence intensity of two cell subsets after PI staining.


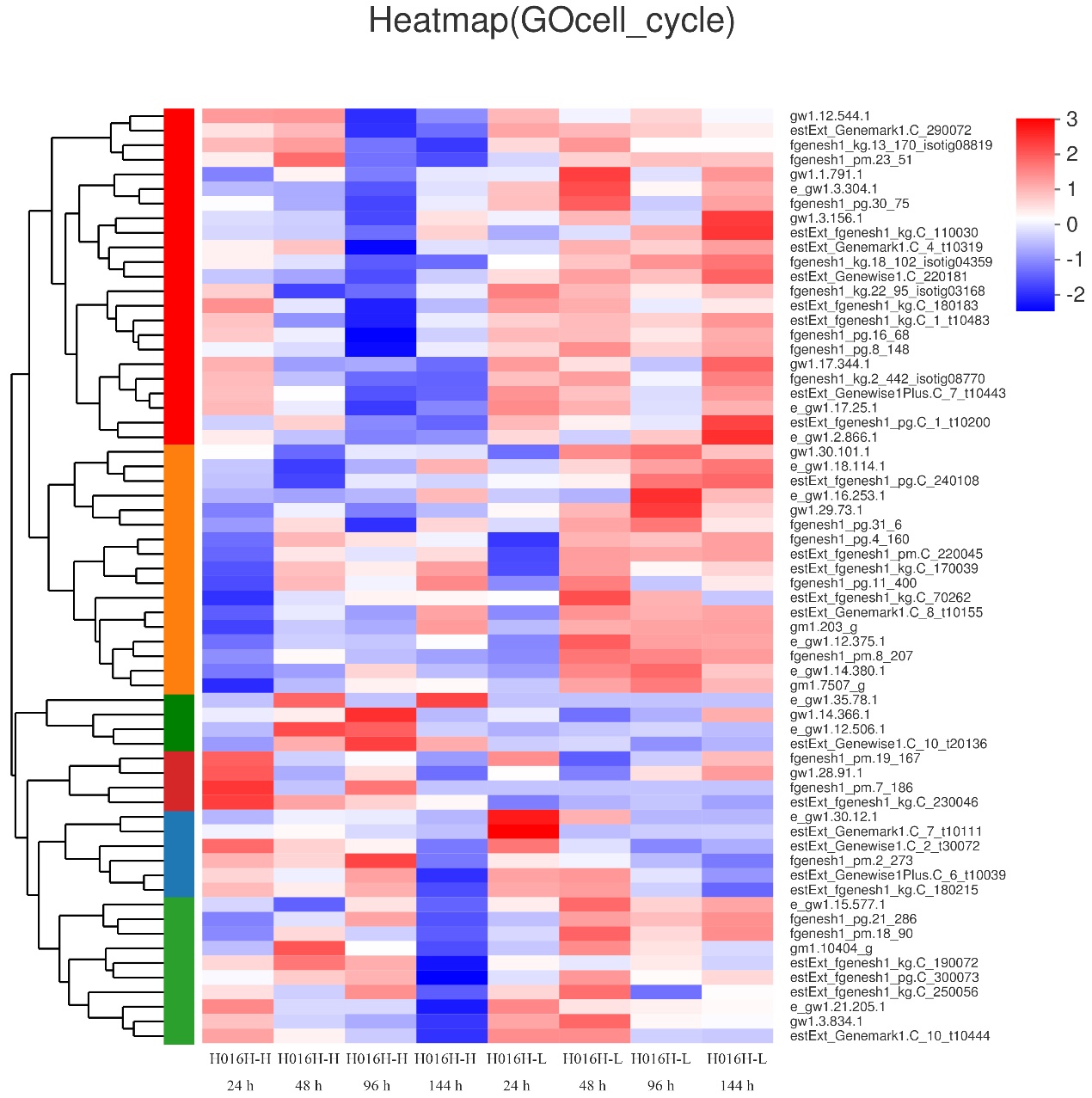


Figure S2. Heatmap showing the relative expression of DEGs related to cell cycle at each stage in H016-H and H016-L.


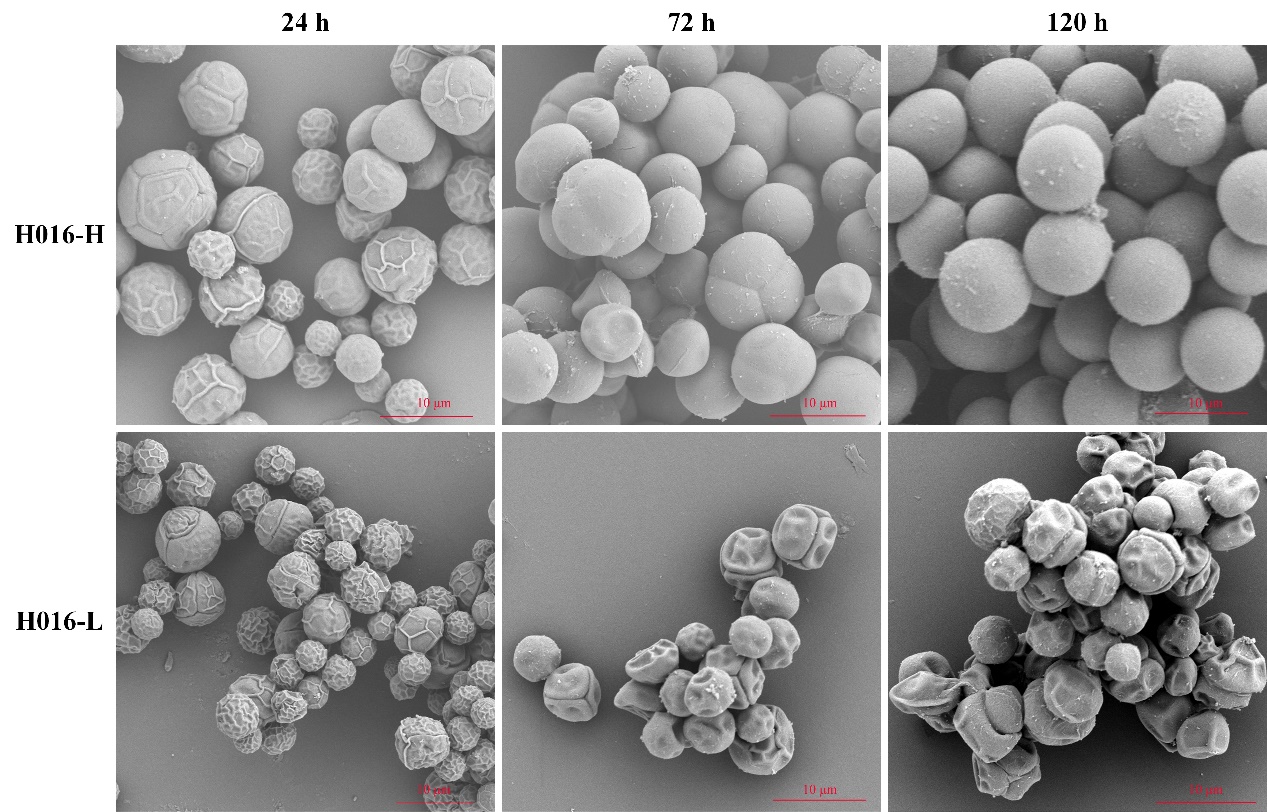


Fig. S3. Scanning electron microscopy observation of cell division status during fermentation process of two cell subpopulations.


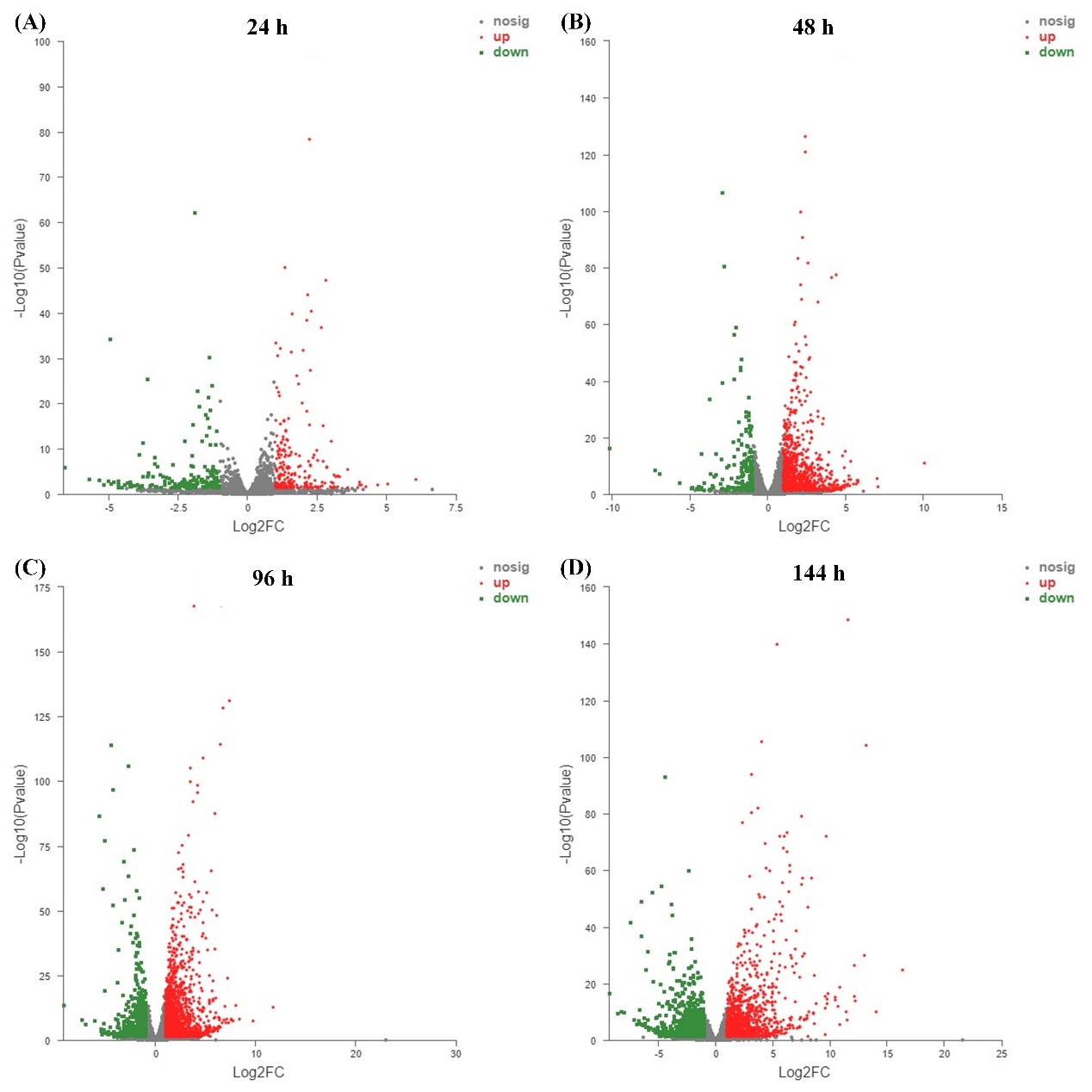


Figure S4. The volcano map of difference expression genes between H016-H and H016-L in fermentation process.


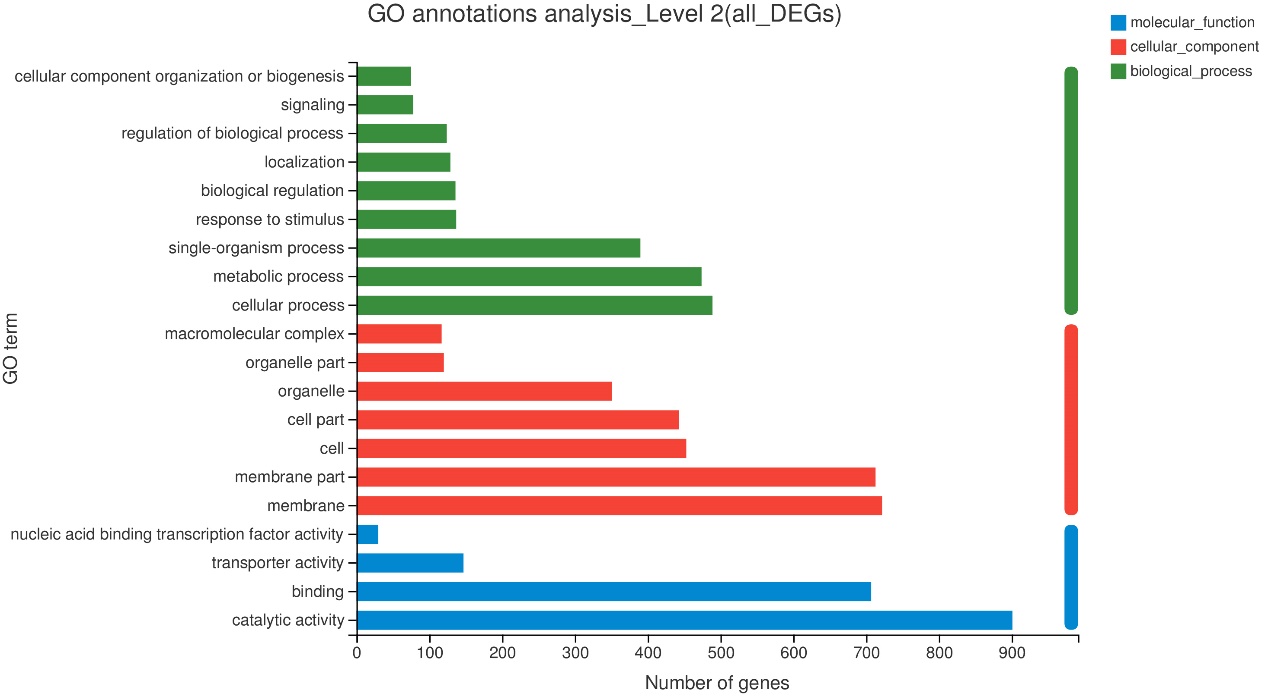


Figure S5. Go classification of difference expression genes between H016-H and H016-L.


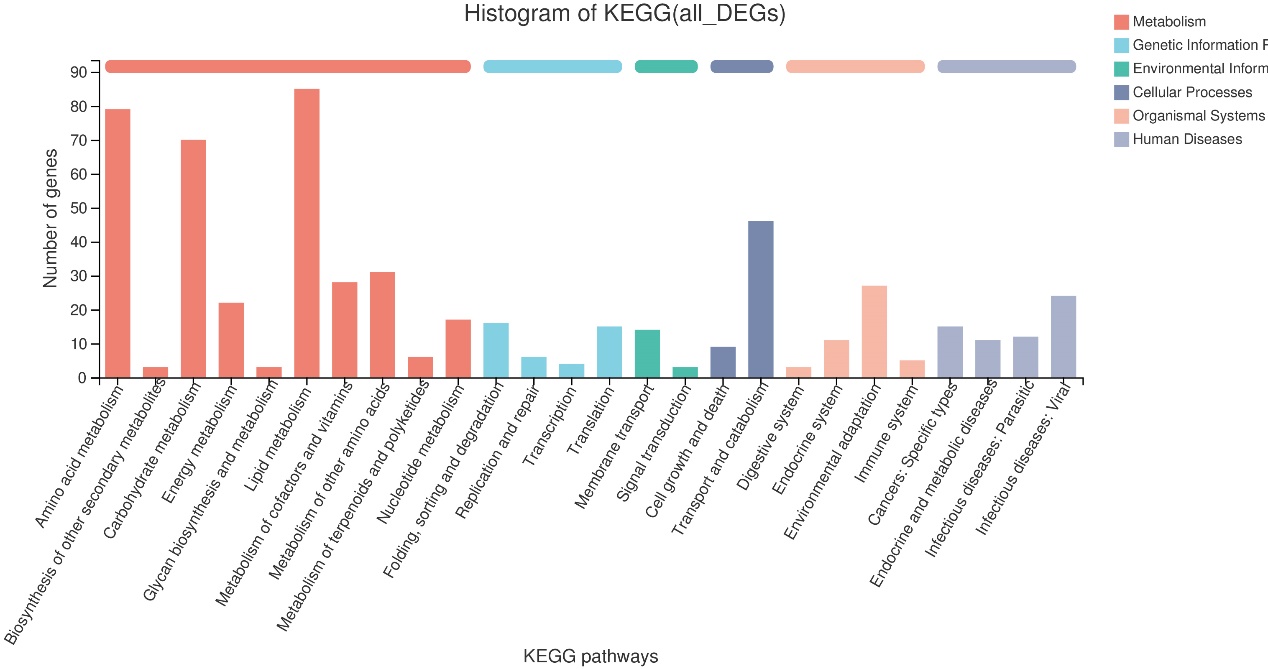


Figure S6. KEGG classification of difference expression genes between H016-H and H016-L.


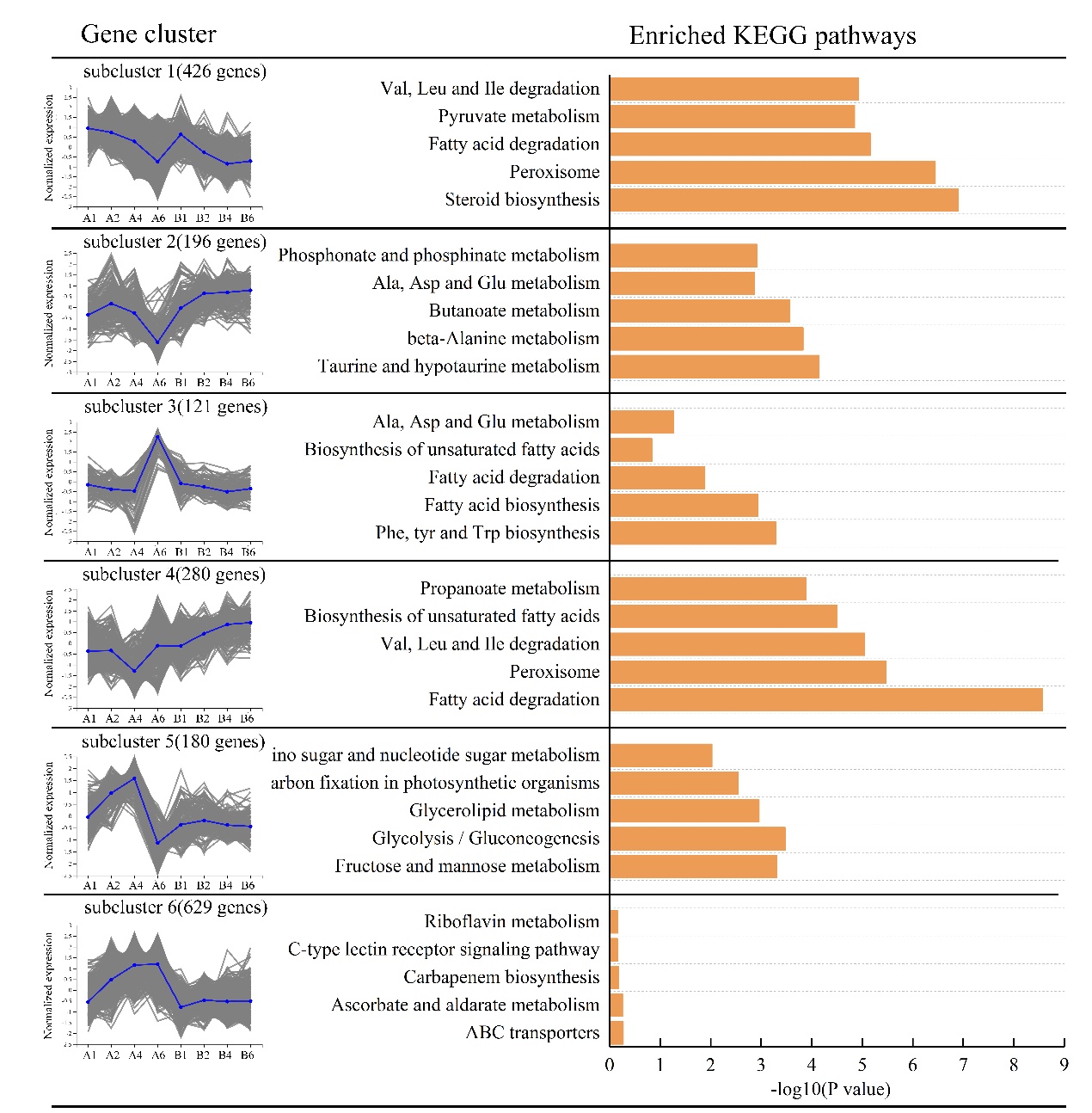


Figure S7. Expression trends of genes in six clusters. The gray lines represent the expression levels of individual genes. The blue lines represent the average expression level of genes in the cluster.


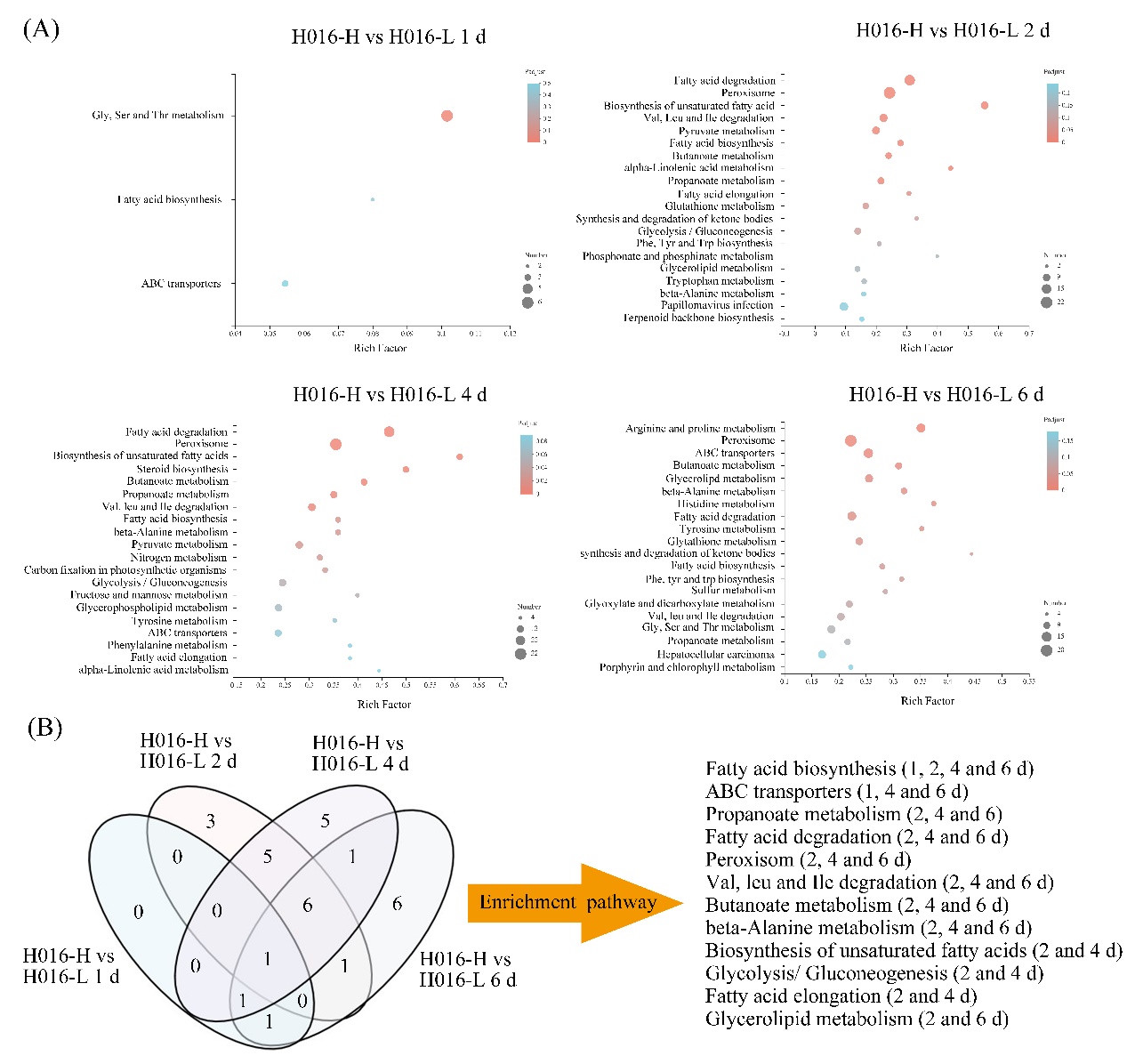


Figure S8. KEGG enrichment analysis of differentially expressed genes in two cell subpopulations during fermentation.
